# Biocompatible Chitosan Films Containing Acetic Acid Manifested Potent Antiviral Activity against Enveloped and Non-Enveloped Viruses

**DOI:** 10.3390/ijms241512028

**Published:** 2023-07-27

**Authors:** Alba Cano-Vicent, Alberto Tuñón-Molina, Miguel Martí, Ángel Serrano-Aroca

**Affiliations:** Biomaterials and Bioengineering Lab, Centro de Investigación Traslacional San Alberto Magno, Universidad Católica de Valencia San Vicente Mártir, 46001 Valencia, Spain; alba.cano@mail.ucv.es (A.C.-V.); alberto.tunon@ucv.es (A.T.-M.); miguel.marti@ucv.es (M.M.)

**Keywords:** chitosan, acetic acid, cytoxicity, antiviral activity, enveloped viruses, non-enveloped viruses

## Abstract

Chitosan films were prepared by solvent casting using an acetic acid-based solution. The films that were developed contained 15.49% of acetic acid solution (10% *v*/*v*) and showed biocompatibility in vitro in human keratinocyte HaCaT cells and potent antiviral activity against both enveloped and non-enveloped viruses. The results showed up to 99.98% and 99.92% viral inactivation against the phi 6 enveloped bacteriophage and MS2 non-enveloped bacteriophage, respectively, suggesting that this chitosan/acetic acid film is a promising material for biomedical applications that require biodegradable broad-spectrum antiviral materials.

## 1. Introduction

While chitin is the major constituent of the exoskeleton of crustaceous water animals, chitosan is a polysaccharide produced by deacetylating chitin [1,2]. Chitosan is inexpensive, renewable, biodegradable and possesses a broad range of biotechnological applications approved by the Food and Drug Administration (FDA) for certain biomedical fields [3]. One of its unique biological properties is its antibacterial activity, which depends on many factors, such as molecular weight and degree of deacetylation [4,5,6]. Chitosan’s polymer structure possesses positive charges that are capable of destroying the cell membrane of a broad range of bacterial species, such as Gram-positive *Staphylococcus aureus* and Gram-negative *Escherichia coli* [6]. 

Chitosan is only soluble in acidic solutions [7], and acetic acid is a naturally occurring liquid organic acid that is transparent, colorless and has a distinctive pungent odor [8]. Acetic acid is an effective disinfectant and is currently used in the food business, in addition to treating ailments brought on by a sedentary lifestyle. Acetic acid has recently been shown to effectively inactivate SARS-CoV-2 with a complete loss of replication [9]. That study showed by transmission electron microscopy that acetic acid disrupts the binding of the SARS-CoV-2 spike protein binding to ACE2, the primary SARS-CoV-2 cell receptor. Acetic acid can therefore be said to be a good natural candidate to dissolve chitosan [8] for developing new potent antiviral materials. 

Chitosan has been studied for its potential antiviral activity against a wide range of viruses, including non-enveloped viruses [10]. Its antiviral capacity has been demonstrated in plants [11] by activating the plant’s immunological system, inducing a defense response [12]. Its antiviral activity has also been shown against the influenza virus [13] or other viruses, such as Herpes [14]. However, its effectiveness as an antiviral agent can depend on various factors, such as the type and concentration of the chitosan used, the virus’s mode of action and the experimental conditions [15]. Chitosan’s antiviral activity may not be effective against all types of enveloped and non-enveloped viruses, and further research is needed to fully understand its potential as an antiviral agent.

In the present study, a chitosan/acetic acid film was produced by the solvent casting method using an acetic acid-based solution. Our aim was to study the material’s biological properties in terms of toxicity in vitro using human keratinocyte HaCaT cells and its antiviral properties against enveloped and non-enveloped viruses. The phi6 bacteriophage can be used as a biosafe viral model of enveloped viruses such as SARS-CoV-2, influenza and Ebola [16], while the MS2 bacteriophage can be used as a biosafe viral model of non-enveloped viruses such as Hepatitis A and Poliovirus Type 1. Therefore, this work aims to develop a new biocompatible material composed of two well-known natural antiviral agents, chitosane and acetic acid, with great promise in biomedicine.

## 2. Results 

### 2.1. Film Composition

The percentage weight of acetic acid in the chitosan films was determined by gravimetric analysis. The results indicated that the %weight of acetic acid solution (10% *v*/*v*) present in the chitosan film was 15.39 ± 1.76. 

### 2.2. Toxicological Study

**Figure 1:**
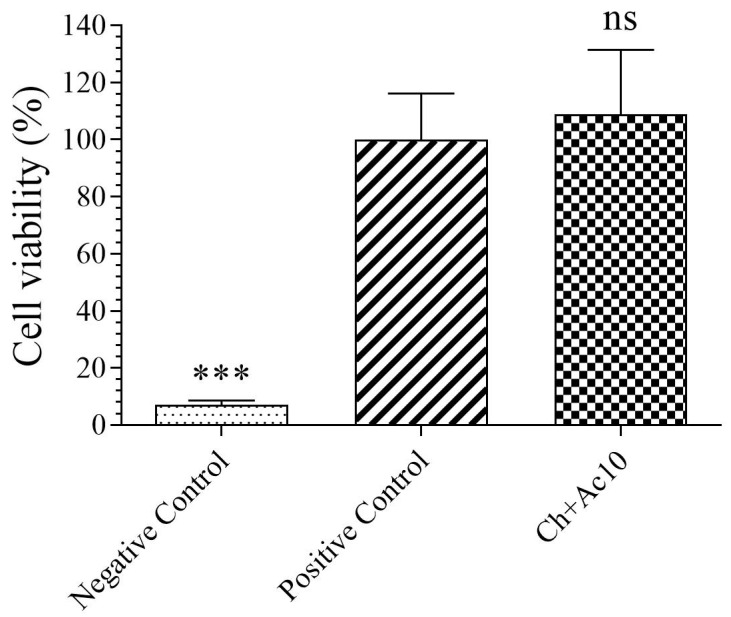
3-[4,5-dimethylthiazol-2-yl]-2,5-diphenyl tetrazolium bromide (MTT) cytotoxicity test of extracts acquired from chitosan/acetic acid film (Ch+Ac10), positive (cells without contact with sample) and negative (cells with zinc solution) controls cultured with human keratinocyte HaCaT cells at 37 °C. *** *p* < 0.001; ns: not significant.

The results of the toxicity assays on the film extract in the presence of human keratinocyte HaCaT cells are shown in Figure 1.

As the sample showed no statistically significant differences in cell viability (%) with respect to those of the positive control, the Ch+Ac10 film was found to be biocompatible in the presence of human keratinocyte cells. 

### 2.3. Antiviral Test against Enveloped and Non-Enveloped Viruses

**Figure 2:**
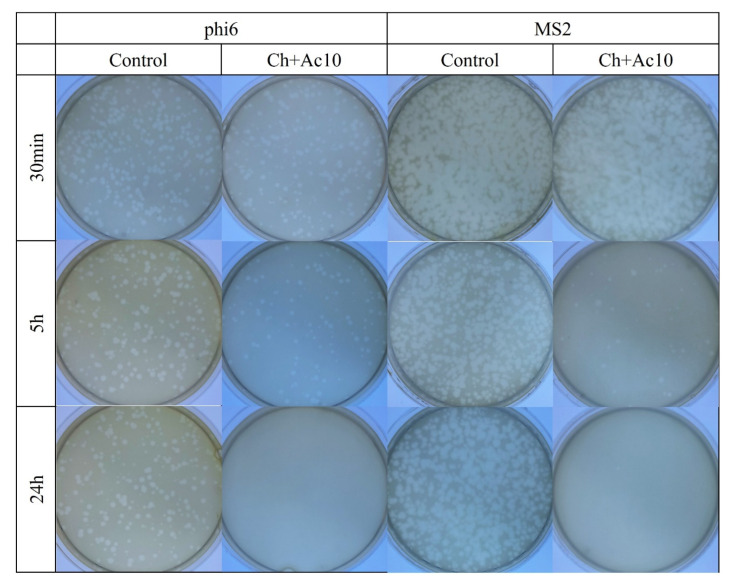
Loss of bacteriophage phi 6 and MS2 viability measured by the double-layer method. Bacteriophage phi 6 and MS2 titration images of 1/100 diluted samples for control and chitosan/acetic acid film (Ch+Ac10) at 30 min, 5 h and 24 h of viral contact.

The results of the antiviral tests showed that chitosan/acetic acid film possesses potent antiviral activity at 5 h and 24 h of viral contact against the enveloped bacteriophage phi6 and the non-enveloped bacteriophage MS2 (Figure 2).

**Figure 3:**
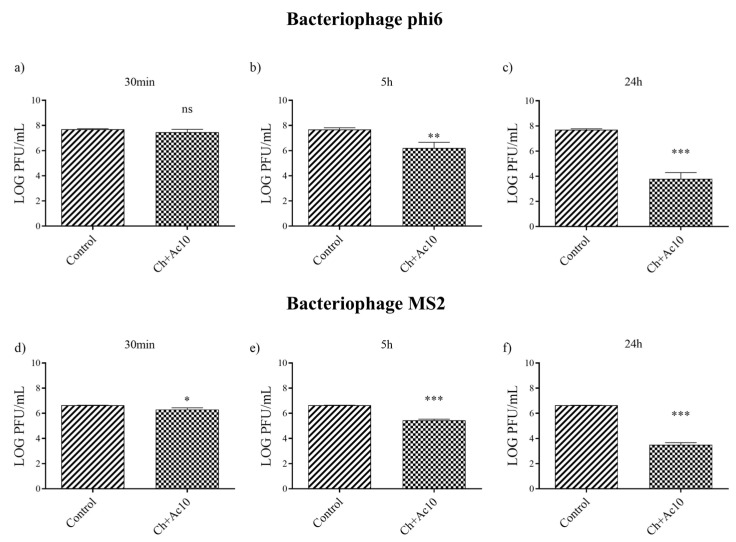
Reduction of infection titers of the phi 6 bacteriophage at 30 min (**a**), 5 h (**b**) and 24 h (**c**) and the MS2 at 30 min (**d**), 5 h (**e**) and 24 h (**f**) in plaque-forming units per mL (PFU/mL) measured by the double-layer method. Control and chitosan/acetic acid film (Ch+Ac10). *** *p* < 0.001; ** *p* < 0.01; * *p* < 0.05; ns, not significant.

After 5 h and 24 h of contact between the Ch+Ac10 film and bacteriophage phi6, bacterial lawns grew in the plate with few plaques (Figure 2), while 30 min of viral contact was not long enough to provide antiviral activity. The antiviral results were quite similar when the film was in contact with the bacteriophage MS2, so that, after 5 h and 24 h of contact, the film showed potent antiviral activity. The plaque-forming units per mL (PFU/mL) of bacteriophage phi6 and bacteriophage MS2 are shown and compared with the control in Figure 3 after being in contact with the Ch+Ac10 film.

After 5 h of viral contact with the bacteriophage phi6 and the bacteriophage MS2, the Ch+Ac10 film inactivated 95.64% and 93.60% of the film, respectively (Table 1).

After 24 h of viral contact between the chitosan/acetic acid film and bacteriophage phi6 and bacteriophage MS2, the % inactivation of the virus was 99.98% and 99.92%, respectively (Table 1), although 30 min of viral contact was not enough to achieve any antiviral effect against either type of virus (Table 1).

### 2.4. Double-Stranded RNA Extraction and Quantification

**Figure 4:**
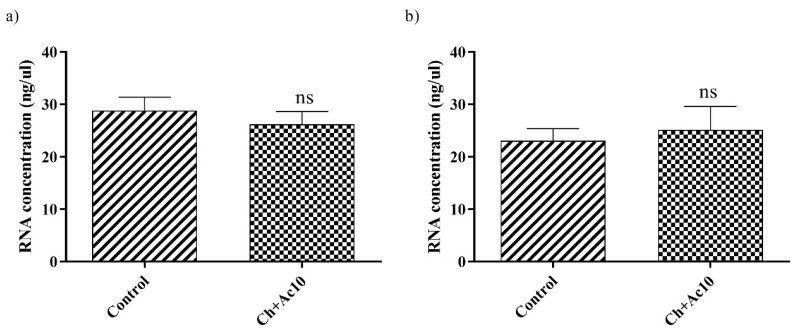
RNA concentration (ng/μL) of bacteriophage phi6 (**a**) and bacteriophage MS2 (**b**) measured in the control (not in contact with the samples) and the same amount of bacteriophage after contact with the chitosan/acetic acid film (Ch+Ac10) film for 24 h; ns, not significant.

RNA extraction and quantification of bacteriophage phi6 and bacteriophage MS2 after being in contact with the Ch+Ac10 film were carried out to show that the virus did not remain adhered to the surface of the film before the antiviral assays, which could have given false results. There were no significant differences in the amount of RNA between the control and the virus that had been in contact with the different samples (Figure 4).

## 3. Discussion

A chitosane film containing 15.39 ± 1.76 of acetic acid solution (10% *v*/*v*) has been developed in this work. This film showed biocompatibility in the presence of human keratinocyte cells. The antiviral tests showed that this chitosan/acetic acid film possesses potent antiviral activity against both the enveloped bacteriophage phi6 and the non-enveloped bacteriophage MS2. The RNA extraction and quantification tests of bacteriophage phi6 and bacteriophage MS2, after being in contact with the Ch+Ac10 film, ensured that the virus did not remain adhered to the surface of the film before the antiviral assays.

Acetic acid is a good candidate as an antiviral agent according to many studies [17,18,19]. Derivates of acetic acid inhibited the replication of enveloped viruses [20,21], and acetic acid inactivates and separates the external glycoproteins of the viral envelope and inhibits the transmission of enveloped viruses [22,23]. This type of acid had a stronger effect with the reduced binding of the spike protein to ACE2, the main SARS-CoV-2 cell receptor [9,24]. In non-enveloped viruses such as bacteriophage MS2 and non-enveloped noroviruses, the mechanism has not been defined but acetic acid could produce physical interactions with the virus to achieve its neutralization [25].

Chitosan can also inhibit viral infections in animal cells, prevent the development of phage infection in infected microbial cultures [13,26] with a mechanism very different from that of acetic acid, increase the immune response and modulate the production of macrophages and granulocytes [27]. However, despite the extensive number of published studies, the exact antiviral mechanism of chitosan and its derivatives is not fully understood [28]. Several antiviral mechanisms proposed for chitosan include direct killing of the virus, electrostatic interaction between the polycationic positive charge of chitosan and the negatively charged surface of the virus disrupting its protective membrane, inhibition of viral adsorption and subsequent host cell invasión, among other mechanisms [13].

Some reports have shown that chitosan has no direct antiviral activity, but instead it stimulates the immune response of the plant and enhances its general defense mechanisms [29,30]. However, other studies showed that low concentrations (0.1 mg/mL) of chitosan can suppress potato virus X and tobacco mosaic virus in a third to a half of treated potato and tomato plants, whereas higher concentrations (1 mg/mL) suppressed the viral infection in 80% of the treated plants [13]. On the other hand, acetic acid is a good candidate as an antiviral agent against enveloped and non-enveloped viruses [9,25]. Chitosan and acetic acid thus make a good combination for combating viral infections as together they inhibit virus replication and stimulate the host’s immune response. Furthermore, this new antiviral hydrogel was found to be biocompatible in the presence of human keratinocyte cells. 

## 4. Materials and Methods

### 4.1. Materials

Chitosan from crab shells (highly viscous, Product number: 48165, Lot # BCBP6349V) was purchased from Sigma-Aldrich (Saint Louis, MO, USA). The chitosan used was previously characterized and showed a molecular weight of 183 kDa [31]. Acetic acid (≥99.8%) was provided by Honeywell through Sigma-Aldrich (Saint Louis, MO, USA). Fetal bovine serum (FBS), DMEM low glucose, penicillin-streptomycin (P/S) and L-glutamine were obtained from Life Technologies (Gibco, Karlsruhe, Germany). Bacteriological agar was purchased from Scharlau (Ferrosa, Barcelona, Spain). Tryptic soy broth (TSB) and tryptic soy agar (TSA) were provided by Liofilchem (Roseto degli Abruzzi, Italy).

### 4.2. Synthesis

Acetic acid was diluted in distilled water to obtain a 10% *v*/*v* solution. A total of 0.25 g of chitosan was dissolved in 30 mL of the acetic acid solution by magnetic stirring for 1 h at 24 ± 0.5 °C. This solution was placed in a Petri dish and the solvent was left to evaporate at room temperature for 24 h to avoid sample cracking. The Petri dish was subsequently left in an oven for 48 h at 37 °C to complete the drying process. This film will be referred hereinafter as Ch+Ac10 film. The amount (n = 6) of acetic acid remaining in the film was determined gravimetrically. Disks of 10 mm diameter were obtained from the films with a sharp cylindrical punch and subjected to ultraviolet radiation for 1 h per side for sterilization.

### 4.3. Toxicological Study

A volume ratio of 3 cm^2^/mL was selected in accordance with ISO-10993, and all the disks were placed in a 6-well plate with DMEM from Life Technologies (Gibco, Karlsruhe, Germany) and no FBS in this concentration. A culture medium was used for growth based on low-glucose DMEM, supplemented with FBS 10%, 1% *w*/*v* penicillin, and 1% *w*/*v* streptomycin. Human keratinocytes HaCaT cells, provided by the La Fe Health Research Institute (Valencia, Spain) were seeded into 96-well plates at a density of 10^4^ cells/well and grown for 24 h at 37 °C in a 5% CO_2_ humidified atmosphere. The medium was then replaced by 100 μL with the corresponding extractions from each sample and incubated for 24 h in the same conditions. The medium was also replaced by 100 μL of the same medium used to produce the film extracts as a positive control. For the negative control, it was replaced by 100 μL of 1000 μM zinc chloride (≥97.0%, Sigma-Aldrich) solution since this concentration is highly toxic for HaCaT cells [32]. Six replicate samples were prepared in wells for each concentration. Cytotoxicity was evaluated by the 3-(4,5-dimethylthiazol-2-yl)-2,5-diphenyl tetrazolium (MTT) assay. MTT was added to replace the cell medium and incubated for 2 h in the same conditions as the culture. Formazan crystals were then solubilized by DMSO, and cell viability was determined from the absorbance at 550 nm on a Varioskan micro plate reader (ThermoScientific, Mississauga, ON, Canada). 

### 4.4. Antiviral Test against Enveloped and Non-Enveloped Viruses

Enveloped bacteriophage phi6 and non-enveloped bacteriophage MS2 infect the Gram-negative *Pseudomonas syringae* and *E. coli*, respectively. The *Pseudomonas syringae* (DSM 21482), the enveloped bacteriophage phi6 (DSM 21518), *E. coli* (DSM 5695) and bacteriophage MS2 (DSM 13767) were obtained from the Leibniz Institute DSMZ-German Collection of Microorganisms and Cell Cultures GmbH (Braunschweig, Germany). *P. syringae* and *E. coli* were first grown on a TSA plate and then in liquid TSB. *P. syringae* was incubated at 25 °C at a speed of 120 rpm, and *E. coli* was incubated at 37 °C at a speed of 120 rpm. The bacteriophages were propagated following the specifications provided by the Leibniz Institute.

The infective activity of the bacteriophages was determined by the double-layer method [33]. A total of 50μL with a titer of approximately 1 × 10^6^ PFU/mL of a bacteriophage suspension in TSB was added to each sample and incubated for 30 min, 5 h and 24 h. The disks with the bacteriophage suspension were placed in a falcon tube with 10 mL of TSB, sonicated for 5 min at 25 °C and subsequently vortexed for 1 min. Serial dilutions were made for each sample. For bacteriophage phi6, 100 μL of *P. syringae* at OD_600nm_ = 0.5 was mixed with 100 μL of each bacteriophage dilution. This suspension was mixed with 4 mL of top agar (TSB + 0.75% bacteriological agar) with 5 mM CaCl_2_ to be finally placed on TSA plates, which were incubated at 25 °C for 24 h. The same procedure was used for bacteriophage MS2, but *E. coli* was at OD_600nm_ = 0.2 and the plates were incubated at 37 °C.

The bacteriophage titer of each sample was calculated and expressed in PFU/mL for comparison with the control, which consisted of the bacteriophage–bacteria suspension that had not been in contact with a disk. The control also confirmed that the sonication/vortexing procedure had no impact on the bacteriophage’s capacity to spread infection. The antiviral tests were carried out in duplicate on two distinct days (n = 6) to ensure reproducible results.

### 4.5. Double-Stranded RNA Extraction and Quantification

Double-stranded RNA extraction and quantification of bacteriophage phi6 and bacteriophage MS2 were carried out to determine whether any viral particles remained attached to the Q+Ac10 sample film and compared to the control before the antiviral assays to avoid false results. A total of 50 μL of the bacteriophage solution at 1 × 10^6^ PFU/mL was dispersed on the disks and incubated for 24 h at 25 °C for the bacteriophage phi6 and at 37 °C for the bacteriophage MS2. A total of 50 μL of the bacteriophage solution that had not been in contact with the samples (control) was incubated in the same conditions. Disks with bacteriophage solution and control were placed in a tube with 10 mL of TSB, sonicated for 5 min and vortexed for 45 s, as in the antiviral assay. RNA was extracted according to the RNA extraction protocol provided by the Norgen Biotek Corp. (Thorold, ON, Canada), [34]. Firstly, a viral particle-lysing was carried out, followed by viral RNA purification. A nanodrop (Thermo Scientific, Waltham, MA, USA) was used to quantity the RNA present in the sample films, and the results were expressed in ng/μL. These measurements were performed in triplicate to analyze reproducibility.

### 4.6. Statistical Analysis

GraphPad Prism 6 software was used to perform one-way analysis of variance for multiple value comparisons followed by Tukey’s post hoc test (* *p* < 0.05, *** *p* < 0.001).

## 5. Conclusions

Chitosan is a cost-effective polysaccharide with favorable biological properties that can be dissolved by acetic acid, a natural antiviral compound. In this study, chitosan films containing 15.39% acetic acid solution (10% *v*/*v*) were prepared by dissolution in acetic acid and subsequent partial evaporation. This novel film showed biocompatibility in vitro in human keratinocyte HaCaT cells. Subsequent antiviral assays showed that this biodegradable material is antiviral against enveloped and non-enveloped viruses. Up to 99.98% of enveloped bacteriophage phi6 and 99.92% of non-enveloped bacteriophage MS2 were inactivated. This chitosan/acetic acid film can therefore be said to be a promising material for advanced applications such as food packaging, wound dressing or drug delivery that require materials capable of preventing broad-spectrum viral infections. 

## Figures and Tables

**Table 1:** Infection titers obtained by the double-layer method for the antiviral assay performed on bacteriophage phi6 and bacteriophage MS2 expressed as mean ± standard deviation, percentage of viral inactivation and log (PFU/mL) reduction with respect to the control after being in contact with the chitosan/acetic acid film (Ch+Ac10) for 30 min, 5 h and 24 h, respectively.

		Bacteriophage phi6		Bacteriophage MS2	
		Control	Ch+Ac10	Control	Ch+Ac10
30 min	PFU/mL	4.96 × 10^7^ ± 5.77 × 10^6^	3.17 × 10^7^ ± 1.37 × 10^7^	4.33 × 10^6^ ± 1.29 × 10^5^	2.08 × 10^6^ ± 6.35 × 10^5^
	log reduction	-	0.23	-	0.33
	% inactivation virus	-	≈0	-	≈0
5 h	PFU/mL	4.91 × 10^7^ ± 1.50 × 10^7^	2.14 × 10^6^ ± 1.59 × 10^6^	4.33 × 10^6^ ± 1.29 × 10^5^	2.77 × 10^5^ ± 6.00 × 10^4^
	log reduction	-	1.47	-	1.20
	% inactivation virus	-	95.64	-	93.60
24 h	PFU/mL	5.12 × 10^6^ ± 3.23 × 10^5^	9.33 × 10^3^ ± 9.45 × 10^3^	4.33 × 10^6^ ± 1.29 × 10^5^	3.33 × 10^3^ ± 1.15 × 10^3^
	log reduction	-	3.91	-	3.13
	% inactivation virus	-	99.98	-	99.92

## Data Availability

Data will be made available on request.
